# Consumption of Olive Oil and Diet Quality and Risk of Dementia-Related Death

**DOI:** 10.1001/jamanetworkopen.2024.10021

**Published:** 2024-05-06

**Authors:** Anne-Julie Tessier, Marianna Cortese, Changzheng Yuan, Kjetil Bjornevik, Alberto Ascherio, Daniel D. Wang, Jorge E. Chavarro, Meir J. Stampfer, Frank B. Hu, Walter C. Willett, Marta Guasch-Ferré

**Affiliations:** 1 Department of Nutrition, Harvard T.H. Chan School of Public Health, Boston, Massachusetts; 2 School of Public Health, the Second Affiliated Hospital, Zhejiang University School of Medicine, Hangzhou, China; 3 Department of Epidemiology, Harvard T.H. Chan School of Public Health, Boston, Massachusetts; 4 Channing Division of Network Medicine, Department of Medicine, Brigham and Women’s Hospital and Harvard Medical School, Boston, Massachusetts; 5 Department of Public Health and Novo Nordisk Foundation Center for Basic Metabolic Research, Faculty of Health and Medical Sciences, University of Copenhagen, Copenhagen, Denmark

## Abstract

**Importance:**

Age-standardized dementia mortality rates are on the rise. Whether long-term consumption of olive oil and diet quality are associated with dementia-related death is unknown.

**Objective:**

To examine the association of olive oil intake with the subsequent risk of dementia-related death and assess the joint association with diet quality and substitution for other fats.

**Design, Setting, and Participants:**

This prospective cohort study examined data from the Nurses’ Health Study (NHS; 1990-2018) and Health Professionals Follow-Up Study (HPFS; 1990-2018). The population included women from the NHS and men from the HPFS who were free of cardiovascular disease and cancer at baseline. Data were analyzed from May 2022 to July 2023.

**Exposures:**

Olive oil intake was assessed every 4 years using a food frequency questionnaire and categorized as (1) never or less than once per month, (2) greater than 0 to less than or equal to 4.5 g/d, (3) greater than 4.5 g/d to less than or equal to 7 g/d, and (4) greater than 7 g/d. Diet quality was based on the Alternative Healthy Eating Index and Mediterranean Diet score.

**Main Outcome and Measure:**

Dementia death was ascertained from death records. Multivariable Cox proportional hazards regressions were used to estimate hazard ratios (HRs) and 95% CIs adjusted for confounders including genetic, sociodemographic, and lifestyle factors.

**Results:**

Of 92 383 participants, 60 582 (65.6%) were women and the mean (SD) age was 56.4 (8.0) years. During 28 years of follow-up (2 183 095 person-years), 4751 dementia-related deaths occurred. Individuals who were homozygous for the apolipoprotein ε4 (*APOE ε4*) allele were 5 to 9 times more likely to die with dementia. Consuming at least 7 g/d of olive oil was associated with a 28% lower risk of dementia-related death (adjusted pooled HR, 0.72 [95% CI, 0.64-0.81]) compared with never or rarely consuming olive oil (*P* for trend < .001); results were consistent after further adjustment for *APOE ε4*. No interaction by diet quality scores was found. In modeled substitution analyses, replacing 5 g/d of margarine and mayonnaise with the equivalent amount of olive oil was associated with an 8% (95% CI, 4%-12%) to 14% (95% CI, 7%-20%) lower risk of dementia mortality. Substitutions for other vegetable oils or butter were not significant.

**Conclusions and Relevance:**

In US adults, higher olive oil intake was associated with a lower risk of dementia-related mortality, irrespective of diet quality. Beyond heart health, the findings extend the current dietary recommendations of choosing olive oil and other vegetable oils for cognitive-related health.

## Key Points

**Question:**

Is the long-term consumption of olive oil associated with dementia-related death risk?

**Findings:**

In a prospective cohort study of 92 383 adults observed over 28 years, the consumption of more than 7 g/d of olive oil was associated with a 28% lower risk of dementia-related death compared with never or rarely consuming olive oil, irrespective of diet quality.

**Meaning:**

These results suggest that olive oil intake represents a potential strategy to reduce dementia mortality risk.

## Teaser

This cohort study investigates the association of olive oil intake with risk of dementia-related death among US adults.

## Introduction

One-third of older adults die with Alzheimer disease or another dementia.^1^ While deaths from diseases such as stroke and heart disease have been decreasing over the past 20 years, age-standardized dementia mortality rates have been on the rise.^2^ The Mediterranean diet has gained in popularity owing to its recognized, multifaceted health benefits, particularly on cardiovascular outcomes.^3^ Accruing evidence suggests this dietary pattern also has a beneficial effect on cognitive health.^4^ As part of the Mediterranean diet, olive oil may exert anti-inflammatory and neuroprotective effects due to its high content of monounsaturated fatty acids and other compounds with antioxidant properties such as vitamin E and polyphenols.^5^ A substudy conducted as part of the Prevencion con Dieta Mediterranea (PREDIMED) randomized trial provided evidence that higher intake of olive oil for 6.5 years combined with adherence to a Mediterranean diet was protective of cognitive decline when compared with a low-fat control diet.^6,7,8^

Given that most previous studies on olive oil consumption and cognition were conducted in Mediterranean countries,^7,8,9,10^ studying the US population, where olive oil consumption is generally lower, could offer unique insights. Recently, we showed that olive oil consumption was associated with a lower risk of total and cause-specific mortality in large US prospective cohort studies, including a 29% (95% CI, 22%-36%) lower risk for neurodegenerative disease mortality in participants who consumed more than 7 g/d of olive oil compared with little or none.^11^ However, this previous analysis was not designed to examine the association of olive oil and diet quality with dementia-related mortality, and therefore the latter remains unclear.

In this study, we examined the association between total olive oil consumption and the subsequent risk of dementia-related mortality in 2 large prospective studies of US women and men. Additionally, we evaluated the joint associations of diet quality (adherence to the Mediterranean diet and Alternative Healthy Eating Index [AHEI] score) and olive oil consumption with the risk of dementia-related mortality. We also estimated the difference in the risk of dementia-related mortality when other dietary fats were substituted with an equivalent amount of olive oil.

## Methods

### Study Population

Analyses were performed in 2 large US prospective cohorts: the Nurses’ Health Study I (NHS) and the Health Professionals Follow-Up Study (HPFS). The NHS was initiated in 1976 and recruited 121 700 US female registered nurses aged 30 to 55 years.^12^ The HPFS was established in 1986 and included 51 525 male health professionals aged 40 to 75 years.^13^ The cohorts have been described elsewhere.^12,13^ Lifestyle factors and medical history were assessed biennially through mailed questionnaires, with a follow-up rate greater than 90%. Baseline for this analysis was 1990, which is when the food frequency questionnaires (FFQs) first included information on olive oil consumption.

Participants with a history of cardiovascular disease (CVD) or cancer at baseline, with missing data on olive oil consumption, or who reported implausible total energy intakes (<500 or >3500 kcal/d for women and <800 or >4200 kcal/d for men) were excluded. The completion of the questionnaire self-selected cognitively highly functioning women and men. In total, 60 582 women and 31 801 men were included. The study protocol was approved by the institutional review boards of the Brigham and Women’s Hospital and Harvard T.H. Chan School of Public Health, which deemed the participants’ completion of the questionnaire to be considered as implied consent. This report followed the Strengthening the Reporting of Observational Studies in Epidemiology (STROBE) reporting guideline.

### Dietary Assessment

Dietary intake was measured using a validated greater than 130-item FFQ administered in 1990 and every 4 years thereafter. The validity and reliability of the FFQ have been described previously.^14^ Participants were asked how frequently they consumed specific foods, including types of fats and oils used for cooking or added to meals in the past 12 months. Total olive oil intake was determined by summing up answers to 3 questions related to olive oil consumption (ie, olive oil used for salad dressings, olive oil added to food or bread, and olive oil used for baking and frying at home). The equivalent of 1 tablespoon of olive oil was considered to be 13.5 g. Intakes of other fats and nutrients were calculated using the United States Department of Agriculture and Harvard University Food Composition Database,^15^ and biochemical analyses. The nutritional composition of olive oil and other types of fat, as well as trends of types of fat intake in the NHS and HPFS, have been reported previously.^11^

Adherence to the Mediterranean diet was assessed using a modified version of the 9-point Alternative Mediterranean index (AMED) score.^16^ Adherence to the AHEI (0-110), previously associated with lower risk of chronic disease, was also computed.^17^ Higher scores indicated better overall diet quality.

### *APOE* Genotyping

The apolipoprotein E ε4 (*APOE ε4*) allele is known to interfere with lipid and glucose metabolism such that it increases the risk of dementia.^18^ *APOE* genotyping was conducted in a subset of 27 296 participants. Blood samples were collected between 1989 and 1990 in the NHS and between 1993 and 1995 in the HPFS. NHS participants who had not provided blood samples were invited to contribute buccal samples from 2002 to 2004. DNA was extracted with the ReturPureGene DNA Isolation Kit (Gentra Systems). The *APOE* genotype was determined using a Taqman Assay (Applied Biosystems)^19^ in 5069 participants, and through imputation from multiple genome-wide association studies,^20^ which has shown high accuracy,^20^ in the remaining subset.

### Ascertainment of Dementia-Related Death

Deaths were ascertained from state vital statistics records and the National Death Index or by reports from next of kin or the postal authorities. The follow-up for mortality exceeded 98% in these cohorts. Dementia deaths were determined by physician review of medical records, autopsy reports, or death certificates. Dementia deaths were those in which dementia was listed as the underlying cause of death, or as a contributing cause of death, or as reported by the family, in the absence of a more likely cause. The *International Classification of Diseases, Eighth Revision (ICD-8)* was used in the NHS and *ICD-9* in the HPFS, which were the revisions used at the inception of those cohorts. Dementia deaths included codes 290.0 (senile dementia, simple type), 290.1 (presenile dementia), and 331.0 (Alzheimer disease). To test the validity of the dementia mortality outcome, we examined the likelihood of dementia mortality by *APOE ε4* allelic dosage (eTable 1 in Supplement 1).^18^ A composite outcome was also created including both participants who reported having dementia during follow-up and later died, with those who had dementia reported on their death certificate.

### Assessment of Covariates

Participants completed biennial questionnaires reporting updates on body weight, smoking, physical activity, multivitamin use, menopausal status, and postmenopausal hormone use in women, family history of dementia, self-report of chronic diseases, and ancestry. History of depression was identified based on antidepressive medication use and self-report of depression. Socioeconomic status (SES) was established through a composite score derived from home address details and various factors such as income, education, and housing; the composite score methods are described in a previous report.^21^ Body mass index (BMI) was obtained by dividing the weight in kilograms by the height in meters squared.

### Statistical Analysis

In each cohort, age-stratified Cox proportional hazard models were used to evaluate the association of olive oil intake with dementia-related mortality. Participant person-time was calculated from baseline until end of follow-up (June 30, 2018, in NHS; January 31, 2018, in HPFS), loss to follow-up, or death, whichever came first. The cumulative average (mean) of olive oil intake from all available FFQs, from baseline until 2014 (or loss to follow-up or death), was used as the exposure. Because potential diet modifications following cancer or CVD diagnosis may not represent long-term diet, we ceased updating dietary variables upon report of these conditions. For missing covariates, we carried forward nonmissing values from previous questionnaires and assigned median values for continuous variables.

Participants were categorized by olive oil intake frequency: never or less than once per month (reference group), greater than 0 to less than or equal to 4.5 g/d, greater than 4.5 g/d to less than or equal to 7 g/d, and greater than 7 g/d. *P* values for linear trends were obtained using the Wald test on a continuous variable represented by the median intake of each category. Multivariable Cox proportional hazard models were used to estimate the hazard ratios (HRs) and 95% CIs for dementia mortality according to categories of olive oil intake, separately in each cohort. Participants were censored at death from causes other than dementia. Model 1 was stratified for age and calendar time. Multivariable model 2 was adjusted for Southern European/Mediterranean ancestry, married, living alone, smoking, alcohol intake, physical activity, multivitamin use, history of hypertension and hypercholesterolemia, in women postmenopausal status and menopausal hormone use, total energy intake, family history of dementia, history of depression, census SES, and BMI. Multivariable model 3 was further adjusted for intake of red meat, fruits and vegetables, nuts, soda, whole grains, and trans-fat, all indicative of diet quality.

In a secondary analysis we used the composite outcome for dementia-related deaths. We also repeated the main analysis in the genotyping subsample. We carried out mediation analyses to calculate the percentage of the association between olive oil intake and dementia-related mortality that is attributable to CVD, hypercholesterolemia, hypertension, and diabetes. We also performed stratified analyses by prespecified subgroups (eMethods in Supplement 1).

A joint analysis was performed to test whether olive oil intake (never or <1/mo, >0 to ≤7g/d, and >7g/d) and the AMED or the AHEI score (tertiles) combined as the exposure was associated with dementia mortality. In substitution analyses, we assessed the risk of dementia-related mortality by replacing 5 g/d of different fat sources, including margarine, mayonnaise, butter, and a combination of other vegetable oils (corn, safflower, soybean, and canola), with olive oil. Both continuous variables as 5-g/d increments were included in a multivariable model 3, mutually adjusted for other types of fat. The difference in the coefficients obtained for olive oil and the substituted fat provided the estimated HR and 95% CI for substituting 5 g/d of olive oil for an equivalent amount of the other fats.

Several exploratory sensitivity analyses were performed including a 4-year lagged analysis, analyses adjusting for other covariates, a cause-specific competing risk model and analyses excluding participants who self-reported having dementia at baseline (n = 12) (eMethods in Supplement 1). Analyses were performed from May 2022 to July 2023 using SAS version 9.4 (SAS Institute). All statistical tests were 2-sided with an α = .05.

## Results

Over 2 183 095 person-years of follow-up, this study documented a total of 4751 dementia deaths (3473 in NHS and 1278 in HPFS; 37 649 total deaths). Among 92 383 participants included at baseline in 1990, 60 582 (65.6%) were women, and the mean (SD) age was 56.4 (8.0) years. Mean (SD) olive oil intake was 1.3 (2.5) g/d in both NHS and HPFS; the mean (SD) adherence score for the Mediterranean diet was 4.5 (1.9) points in the NHS and 4.2 (1.9) points in the HPFS; and the mean (SD) AHEI diet quality score was 52.5 (11.1) points in the NHS and 53.4 (11.6) points in the HPFS.

Table 1 shows baseline characteristics of participants categorized by total olive oil intake. Participants with a higher olive oil intake (>7 g/d) at baseline had an overall higher caloric intake, but not a higher BMI, had better diet quality, had higher alcohol intake, were more physically active, and were less likely to smoke compared with those never consuming olive oil or less than once per month (Table1). Individuals who were homozygous for the *APOE ε4* allele were 5.5 to 9.4 times more likely to die with dementia compared with noncarriers for the APOE e4 allele (χ^2^ *P* < .001) (eTable 1 in Supplement 1).

**Table 1.** Age-Standardized Baseline Characteristics of Participants by Cohorts^a^

	Olive oil intake			
	Never or <1/mo	>0-≤4.5 g/d	>4.5-≤7 g/d	>7 g/d
**NHS**				
No. of participants	32 360	22 684	2393	3145
Total olive oil, g/d	0	1.5 (1.2)	5.8 (0.5)	9.0 (4.7)
Age, y	56.2 (7.2)	56.1 (7)	56.3 (7)	56.5 (7)
BMI	25.8 (5)	25.4 (4.7)	25.3 (4.5)	25.3 (4.6)
Hypertension, %	18.3	18.3	17.2	17.6
Hypercholesterolemia, %	28.3	31.2	31.3	32.6
Diabetes, %	3.1	2.4	2.3	2.7
Family history of dementia, %	20.5	20.1	19.8	20.5
Current smoker, %	16.3	16.6	15.5	12.8
Physical activity, MET-h/wk	14.4 (19.8)	16.5 (22.9)	18.3 (23.2)	18.8 (25.9)
Total calories, kcal/d	1702 (497)	1762 (501)	1907 (512)	1989 (531)
Alcohol intake, g/d	4.1 (8.6)	6 (10.2)	7.3 (11)	7.2 (10.9)
AMED score, 0-9	4.1 (1.8)	4.8 (1.8)	5.5 (1.7)	5.7 (1.7)
AHEI score, 0-110	50.8 (10.9)	53.8 (10.8)	56.8 (10.6)	58.1 (10.7)
Red and processed meat, servings/d	0.9 (0.6)	0.9 (0.6)	0.8 (0.6)	0.8 (0.6)
Fruits and vegetables, servings/d	4.8 (1.9)	5.3 (2)	6 (2.1)	6.4 (2.2)
Whole grains, servings/d	1.8 (1.5)	1.9 (1.6)	2.2 (1.7)	2.3 (1.8)
Total nuts, servings/d	0.1 (0.2)	0.1 (0.2)	0.2 (0.2)	0.2 (0.2)
Soda, servings/d	0.8 (0.9)	0.8 (0.9)	0.7 (0.8)	0.8 (0.9)
Butter, g/d	1.1 (2.9)	1.3 (3.1)	1.6 (3.3)	1.7 (3.8)
Mayonnaise, g/d	5.5 (7)	4.6 (5.5)	5.2 (6.7)	6.5 (9)
Other vegetable oils, g/d	4.6 (4.3)	4.2 (3.5)	5.1 (4.3)	6.6 (6)
Margarine, g/d	14.7 (17.1)	14.0 (16.6)	13.8 (17)	14.7 (17.6)
Total fat, % kcal [g/d]	31.5 [59.8 (21.9)]	31.0 [60.8 (21.5)]	32.1 [67.8 (22.1)]	33.5 [74 (24.8)]
Saturated fat, % kcal [g/d]	10.9 [20.7 (8.1)]	10.5 [20.6 (8)]	10.2 [21.7 (8.2)]	10.1 [22.5 (8.6)]
Trans-fat, % kcal [g/d]	1.6 [3.0 (1.5)]	1.4 [2.8 (1.4)]	1.3 [2.8 (1.4)]	1.3 [2.9 (1.4)]
Polyunsaturated fat, % kcal [g/d]	5.9 [11.2 (4.6)]	5.8 [11.4 (4.4)]	6.1 [12.9 (4.8)]	6.7 [14.7 (6.1)]
Monounsaturated fat, % kcal [g/d]	11.9 [22.6 (8.7)]	12.0 [23.5 (8.6)]	13.0 [27.3 (8.7)]	13.9 [30.6 (10.2)]
**HPFS**				
No. of participants	16 075	12 855	1246	1625
Total olive oil, g/d	0 (0)	1.5 (1.1)	5.8 (0.5)	9.1 (4.5)
Age, y	57 (9.7)	56.4 (9.2)	57.2 (9)	57.3 (9.1)
BMI	25.6 (3.4)	25.5 (3.2)	25.5 (3.1)	25.3 (3.2)
Hypertension, %	16.2	17.6	16.5	16.9
Hypercholesterolemia, %	19.1	20.9	21.7	23.4
Diabetes, %	2.8	2.7	2.7	2.7
Family history of dementia, %	15.9	16.0	17.4	16.9
Current smoker, %	8.2	7.3	7.4	5.3
Physical activity, MET-h/wk	36.6 (42.6)	37.8 (39.5)	40.4 (37.4)	43.6 (45.4)
Total calories, kcal/d	1897 (577)	1927 (582)	2065 (605)	2134 (605)
Alcohol intake, g/d	8.4 (13.5)	11.2 (14.6)	12.8 (15)	13.3 (15)
AMED score, 0-9	3.8 (1.8)	4.5 (1.9)	5.2 (1.8)	5.5 (1.7)
AHEI score, 0-110	51.1 (11.5)	54.8 (11.1)	58.3 (11.6)	60 (11.4)
Red and processed meat, servings/d	1.1 (0.8)	1.0 (0.8)	1.0 (0.8)	0.8 (0.7)
Fruits and vegetables, servings/d	5.2 (2.4)	5.7 (2.4)	6.6 (2.7)	7.2 (2.9)
Whole grains, servings/d	1 (0.9)	1 (0.9)	1.1 (0.9)	1.2 (1.1)
Total nuts, servings/d	0.2 (0.3)	0.3 (0.3)	0.3 (0.4)	0.3 (0.5)
Soda, servings/d	0.8 (0.9)	0.7 (0.9)	0.7 (0.9)	0.7 (0.9)
Butter, g/d	1.2 (3.3)	1.4 (3.3)	1.5 (3.3)	1.6 (3.6)
Mayonnaise, g/d	4.8 (6.7)	3.9 (5.5)	4.3 (6.4)	4.3 (6.9)
Other vegetable oils, g/d	4.5 (4.3)	4.1 (3.7)	4.8 (4.3)	5.4 (5)
Margarine, g/d	12.6 (16.5)	10.8 (14.8)	10.7 (15.3)	10.1 (14.7)
Total fat, % kcal [g/d]	31.3 [66.5 (26.4)]	30.5 [65.9 (25.5)]	31.5 [72.6 (27.2)]	31.7 [75.3 (27)]
Saturated fat, % kcal [g/d]	10.6 [22.5 (9.8)]	10.1 [21.8 (9.4)]	9.8 [22.7 (9.8)]	9.4 [22.5 (9.6)]
Trans-fat, % kcal [g/d]	1.6 [3.5 (1.9)]	1.5 [3.2 (1.7)]	1.3 [3.1 (1.8)]	1.2 [2.9 (1.6)]
Polyunsaturated fat, % kcal [g/d]	5.9 [12.4 (5.3)]	5.8 [12.5 (5)]	6.1 [14.0 (5.6)]	6.3 [15.0 (6.2)]
Monounsaturated fat, % kcal [g/d]	12.1 [25.8 (10.8)]	12.0 [25.9 (10.4)]	13.0 [29.8 (11.2)]	13.4 [31.6 (11.3)]

Abbreviations: AHEI, alternative healthy eating index; AMED, alternative Mediterranean diet index; BMI, body mass index (calculated as weight in kilograms divided by height in meters squared); HPFS, Health Professionals’ Follow-up Study; MET-h/wk, metabolic equivalent of task–hour per week; NHS, Nurses’ Health Study I.

^a^ Values are means (SD) for continuous variables and No. (%) for categorical variables.

Olive oil intake was inversely associated with dementia-related mortality in age-stratified and multivariable-adjusted models (Table 2). Compared with participants with the lowest olive oil intake, the pooled HR for dementia-related death among participants with the highest olive oil intake (>7 g/d) was 0.72 (95% CI, 0.64-0.81), after adjusting for sociodemographic and lifestyle factors. The association between each 5-g increment in olive oil consumption with dementia-related death was also inverse and significant in the pooled analysis. The multivariable-adjusted HR for dementia-related death for the highest compared with the lowest olive oil intake (>7 g/d) was 0.67 (95% CI, 0.59-0.77) for women and 0.87 (95% CI, 0.69-1.09) for men (Table 2). Olive oil intake in 5-g increments was inversely associated with dementia-related mortality in women (HR, 0.88 [95% CI, 0.84-0.93]), but not in men (HR, 0.96 [95% CI, 0.88-1.04]). Analyses remained consistent when using the composite outcome for death with dementia (eTable 2 in Supplement 2). In the genotyping subsample, the results remained unchanged after further adjusting for the *APOE ε4* allelic genotype (multivariable-adjusted pooled HR comparing high vs low olive oil intake, 0.66 [95% CI, 0.54-0.81]; *P* for trend < .001) (eTable 4 in Supplement 1). Pooled mediation analyses found that CVD, hypercholesterolemia, hypertension, and diabetes did not significantly attenuate the association (unchanged HRs with and without adjusting for the intermediate; data not shown).

**Table 2.** Risk of Dementia-Related Mortality According to Categories of Total Olive Oil

	Category of cumulative average olive oil intake, HR (95% CI)				*P* for trend	HR (95% CI) for 5 g increase in olive oil intake
	Never or <1/mo	>0 to ≤4.5 g/d	>4.5 to ≤7 g/d	>7 g/d		
NHS						
Total olive oil, mean (SD)	0	1.5 (1.2)	5.6 (0.7)	11.9 (5.8)	NA	NA
No. of cases/person-years	1088/438 566	1829/739 116	250/125 884	306/182 726	NA	NA
Age-adjusted model 1	1 [Reference]	0.69 (0.64-0.75)	0.66 (0.57-0.75)	0.53 (0.47-0.60)	<.001	0.84 (0.80-0.88)
Multivariable model 2	1 [Reference]	0.86 (0.80-0.93)	0.85 (0.74-0.98)	0.69 (0.60-0.79)	<.001	0.89 (0.85-0.94)
Multivariable model 3	1 [Reference]	0.86 (0.79-0.93)	0.82 (0.71-0.95)	0.67 (0.59-0.77)	<.001	0.88 (0.84-0.93)
HPFS						
Total olive oil, mean (SD)	0	1.5 (1.2)	5.6 (0.7)	11.4 (5.5)	NA	NA
No. cases/person-years	407/226 931	681/381 397	86/57 337	104/72 138	NA	NA
Age-adjusted model 1	1 [Reference]	0.84 (0.74-0.95)	0.71 (0.56-0.90)	0.65 (0.52-0.80)	<.001	0.87 (0.80-0.95)
Multivariable model 2	1 [Reference]	0.97 (0.85-1.10)	0.87 (0.69-1.11)	0.84 (0.67-1.05)	.09	0.95 (0.87-1.03)
Multivariable model 3	1 [Reference]	0.98 (0.86-1.12)	0.90 (0.71-1.15)	0.87 (0.69-1.09)	.17	0.96 (0.88-1.04)
Pooled data						
Multivariable model 3	1 [Reference]	0.88 (0.83-0.94)	0.84 (0.75-0.95)	0.72 (0.64-0.81)	<.001	0.91 (0.87-0.94)
Meta-analysis						
Multivariable model 3	1 [Reference]	0.89 (0.83-0.95)	0.84 (0.75-0.95)	0.72 (0.64-0.81)	<.001	0.90 (0.87-0.94)

Abbreviations: NHS, Nurses’ Health Study I; HPFS, Health Professionals Follow-up Study.

All Cox proportional hazards models were stratified by age and calendar time. Multivariable model 2 was adjusted for Southern European/Mediterranean ancestry (yes/no), married (yes/no), living alone (yes/no), smoking status (never, former, current smoker 1-14 cigarettes/d, 15-24 cigarettes/d, or ≥25 cigarettes/d), alcohol intake (0, 0.1-4.9, 5.0-9.9, 10.0-14.9, and ≥15.0 g/d), physical activity (<3.0, 3.0-8.9, 9.0-17.9, 18.0-26.9, ≥27.0 metabolic equivalent of task–h/wk), multivitamin use (yes/no), history of hypertension (yes/no), history of hypercholesterolemia (yes/no), history of diabetes (yes/no), in women postmenopausal status and menopausal hormone use (premenopausal, postmenopausal [no, past, or current hormone use]), total energy intake (kcal/d), family history of dementia (yes/no), history of depression (yes/no), census socioeconomic status (9-variable score, in quintiles), and body mass index calculated as weight in kilograms divided by height in meters squared (<23, 23-25, 25-30, 30-35, ≥35). Multivariable model 3 was additionally adjusted for red meat, fruits and vegetables, nuts, soda, whole grains intake (in quintiles), and trans-fat (% kcal). Pooled results were obtained by pooling the datasets of the cohorts, and Cox proportional hazards model 3 was further stratified by cohort (sex). No significant cohort by olive oil intake interaction was found. In a secondary analysis, results were meta-analyzed using a fixed-effect inverse variance-weighted approach. No significant heterogeneity between cohorts was found.

**Figure 1.**
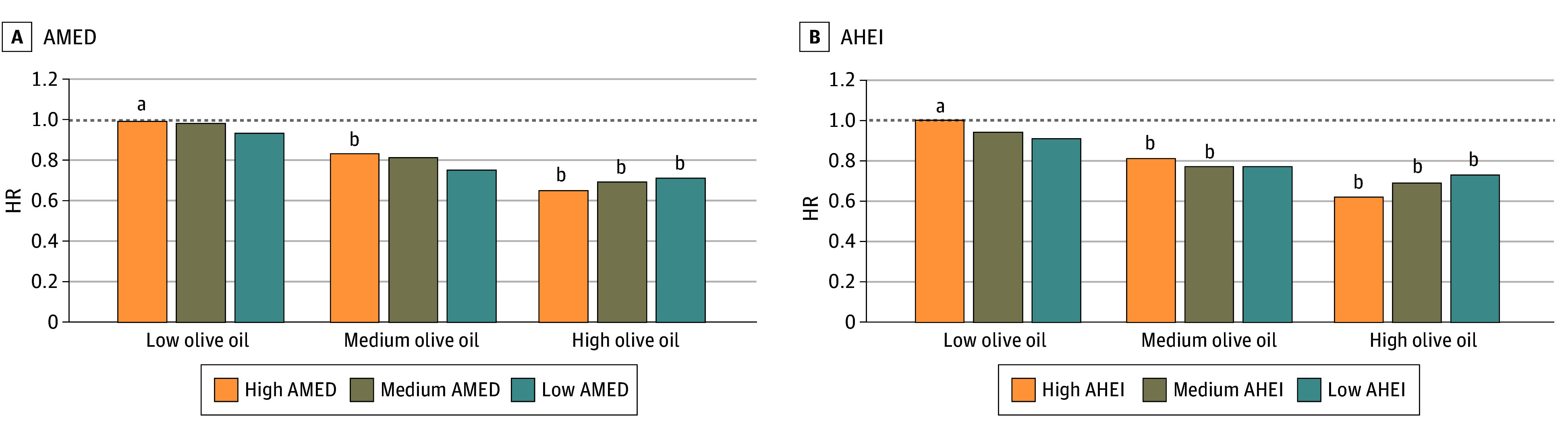
Joint Associations of Olive Oil Intake and Alternative Mediterranean Diet Index (AMED) and Alternative Healthy Eating Index (AHEI) With Dementia-Related Mortality Models were stratified by age, cohort (sex), and calendar time, and adjusted for Southern European/Mediterranean ancestry (yes/no), married (yes/no), living alone (yes/no), smoking status (never, former, current smoker 1-14 cigarettes/d, 15-24 cigarettes/d, or ≥25 cigarettes/d), physical activity (<3.0, 3.0-8.9, 9.0-17.9, 18.0-26.9, ≥27.0 metabolic equivalent of task–h/wk), multivitamin use (yes/no), history of hypertension (yes/no), history of hypercholesterolemia (yes/no), history of diabetes (yes/no), in women postmenopausal status and menopausal hormone use (premenopausal, postmenopausal [no, past, or current hormone use]), total energy intake (kcal/d), family history of dementia (yes/no), history of depression (yes/no), census socioeconomic status (9-variable score, in quintiles), and body mass index calculated as weight in kilograms divided by height in meters squared (<23, 23-25, 25-30, 30-35, ≥35). Pooled results were obtained by pooling the datasets of the cohorts. AMED score is without monounsaturated:saturated fats intake ratio component. AHEI score is without polyunsaturated fats intake component. HR indicates hazard ratio. ^a^Reference value. ^b^*P* < .05.

In joint analyses, participants with the highest olive oil intake had a lower risk for dementia-related mortality, irrespective of their AMED score (28% to 34% lower risk compared with participants in the combined low olive oil and high AMED) (Figure 1A; eTable 3 in Supplement 1) and of their AHEI (27% to 38% lower risk compared with participants with low olive oil and high AHEI) (Figure 1B; eTable 3 in Supplement 1).

**Figure 2.**
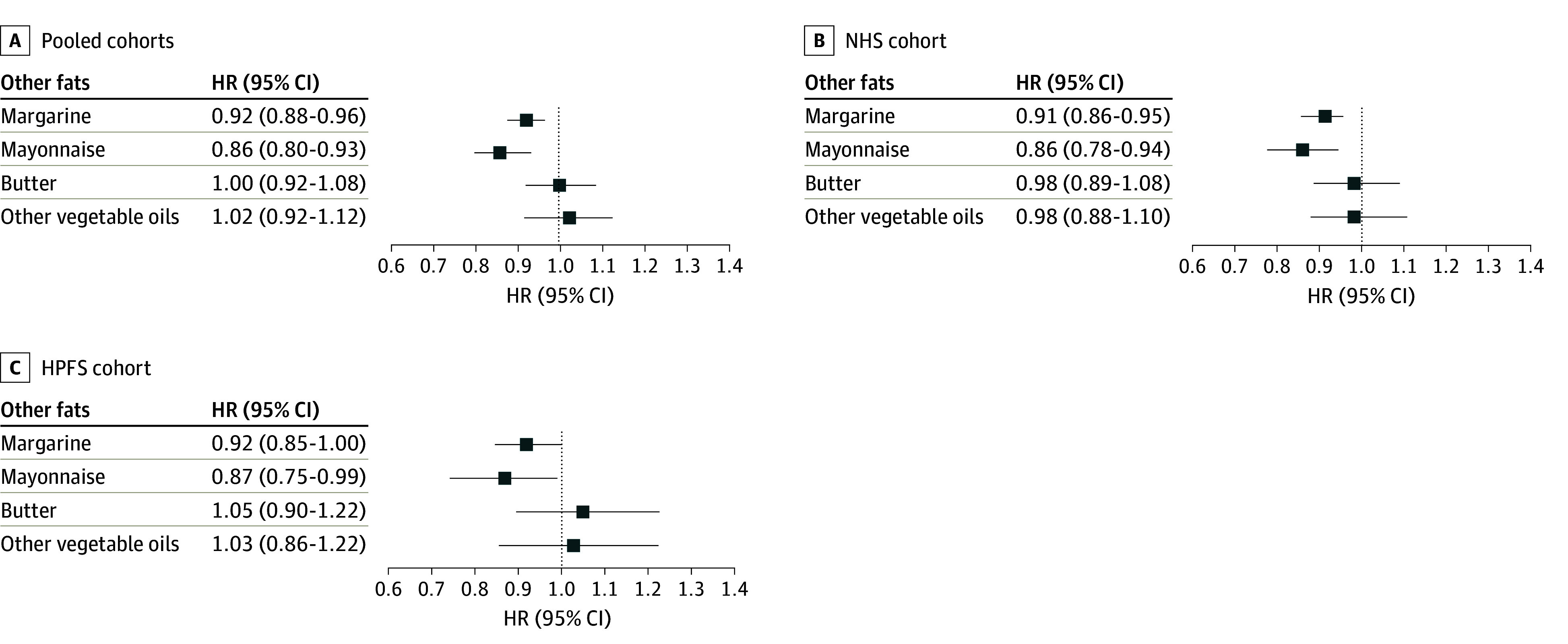
Substitution of Olive Oil for Other Fats Associated With Dementia-Related Mortality Risk in Pooled Cohorts, Nurses’ Health Study (NHS), and Health Professionals’ Follow-Up Study (HPFS) Substitution analysis of 5 g/d intake of olive oil for the equivalent amount of butter, other vegetable oils, mayonnaise, and margarine. All Cox proportional hazards models were stratified by age and calendar time. Models were adjusted for Southern European/Mediterranean ancestry (yes/no), married (yes/no), living alone (yes/no), smoking status (never, former, current smoker 1-14 cigarettes/d, 15-24 cigarettes/d, or ≥25 cigarettes/d), alcohol intake (0, 0.1-4.9, 5.0-9.9, 10.0-14.9, and ≥15.0 g/d), physical activity (<3.0, 3.0-8.9, 9.0-17.9, 18.0-26.9, ≥27.0 metabolic equivalent of task–h/wk), multivitamin use (yes/no), history of hypertension (yes/no), history of hypercholesterolemia (yes/no), in women postmenopausal status and menopausal hormone use (premenopausal, postmenopausal [no, past, or current hormone use]), total energy intake (kcal/d), family history of dementia (yes/no), history of depression (yes/no), census socioeconomic status (9-variable score, in quintiles), body mass index calculated as weight in kilograms divided by height in meters squared (<23, 23-25, 25-30, 30-35, ≥35), red meat, fruits and vegetables, nuts, soda, whole grains intake (in quintiles), and trans-fat. Pooled results were obtained by pooling the data sets of the cohorts and Cox proportional hazards model 3 was further stratified by cohort (sex). HR indicates hazard ratio.

Replacing 5 g/d of mayonnaise with 5 g/d of olive oil was associated with a 14% (95% CI, 7%-20%) lower risk of dementia-related mortality in pooled multivariable-adjusted models (Figure 2). As for the substitution of 5 g/d of margarine with the equivalent amount of olive oil, we estimated an 8% (95% CI, 4%-12%) lower risk. Substitutions of other vegetable oils or butter with olive oil were not statistically significant.

Exploratory subgroup analyses (eFigure in Supplement 1) showed associations between higher olive oil intake and lower risk of dementia-related mortality across most subgroups. No statistically significant associations were found in participants with a family history of dementia, living alone, using a multivitamin, and in non–*APOE ε4* carriers. Results from exploratory sensitivity analyses (eTables 5-8 in Supplement 1) were comparable with the findings from the main analysis (eResults in Supplement 1).

## Discussion

In 2 large US prospective cohorts of men and women, we found that participants who consumed more than 7 g/d of olive oil had 28% lower risk of dying from dementia compared with participants who never or rarely consumed olive oil. This association remained significant after adjustment for diet quality scores including adherence to the Mediterranean diet. We estimated that substituting 5 g/d of margarine and mayonnaise with olive oil was associated with significantly lower dementia-related death risk, but not when substituting butter and other vegetable oils. These findings provide evidence to support dietary recommendations advocating for the use of olive oil and other vegetable oils as a potential strategy to maintain overall health and prevent dementia.

In the NHS and HPFS, a lower risk of neurodegenerative disease mortality, including dementia mortality, was observed with higher olive oil consumption (HR, 0.81 [95% CI, 0.78-0.84]).^11^ Evidence that pertains to cognitive decline or incident dementia is more widely available than it is for dementia mortality.^6,22^ In the French Three-City Study (n = 6947), participants with the highest olive oil intake were 17% (95% CI, 1%-29%) less likely to experience a 4-year cognitive decline related to visual memory, but no association was found for verbal fluency (odds ratio [OR], 0.85 [95% CI, 0.70-1.03]).^22^ Furthermore, participants with a higher intake of olive oil (moderate or intensive vs never) had a lower risk of verbal fluency and visual memory cognitive impairment. Potential sex differences were not investigated. In the PREDIMED trial, which supplemented a Mediterranean-style diet with extra-virgin olive oil (1 L/wk/household) or nuts (30 g/d),^23^ the authors investigated cognitive effects and status in 285 and 522 cognitively healthy participants using global and in-depth neuropsychological battery testing. Although the study was not originally designed for cognitive outcomes and the effect of olive oil cannot be isolated, after 6.5 years, the olive oil group exhibited improved cognitive performance in verbal fluency and memory tests compared with a low-fat diet (control), and they were less prone to develop mild cognitive impairment (OR, 0.34 [95% CI, 0.12-0.97]; n = 285).^6^ Global cognitive performance was higher in both the olive oil and the nut groups compared with the control post trial (n = 522).^8^ These studies were conducted in Europe, in populations with typically higher olive oil intake compared with US populations.

Observational studies and some trials have consistently found associations between following diets such as the Mediterranean, DASH, MIND, and AHEI, and prudent patterns to healthier brain structure,^24^ reduced cognitive impairment and Alzheimer risk, and improved cognitive function.^4^ In our study, those with the highest olive oil intake (>7 g/d) had the lowest dementia-related death risk compared with those with minimal intake (never or less than once per month), regardless of diet quality. This highlights a potentially specific role for olive oil. Still, the group with both high AHEI scores and high olive oil intake exhibited the lowest dementia mortality risk (HR, 0.68 [95% CI, 0.58-0.79]; reference: low AHEI score and low olive oil intake), suggesting that combining higher diet quality with higher olive oil intake may confer enhanced benefit.

Olive oil consumption may lower dementia mortality by improving vascular health.^18^ Several clinical trials support the effect of olive oil in reducing CVD via improved endothelial function, coagulation, lipid metabolism, oxidative stress, platelet aggregation and decreased inflammation.^25^ Nonetheless, the results of our study remained independent of hypertension and hypercholesterolemia. Mild cognitive impairment, Alzheimer disease, and related dementias were associated with abnormal blood brain barrier permeability, possibly allowing the crossing of neurotoxic molecules into the brain.^26^ Mechanistical evidence from animal^27,28,29^ and human studies^9,30^ have shown that phenolic compounds in olive oil, particularly extra-virgin olive oil, may attenuate inflammation, oxidative stress and restore blood brain barrier function, thereby reducing brain amyloid-β and tau-related pathologies and improving cognitive function. However, incident CVD, hypercholesterolemia, hypertension, and diabetes were not significant mediators of the association between olive oil intake and dementia-related death in our study.

The association was significant in both sexes but did not remain in men after full adjustment of the model. Some previous research has reported cognitive-related sex differences. Evidence from trials also showed sex- and/or gender-specific responses to lifestyle interventions for preventing cognitive decline, possibly due to differences in brain structure, hormones (sex) and social factors (gender).^31^ Olive oil intake may be protective of dementia and related mortality, particularly in women. Nonetheless, we did not observe significant heterogeneity or interaction of cohort by olive oil intake on the risk of fatal dementia. Sex and gender differences should be carefully considered in future studies examining the association or effect of olive oil on cognitive-related outcomes to improve our understanding.

We found that using olive oil instead of margarine and mayonnaise, but not butter and other vegetable oils, was associated with a lower risk of dementia-related death. At the time of the study, margarine and mayonnaise contained considerable levels of hydrogenated trans-fats. The latter were strongly associated with all-cause mortality, CVD, type 2 diabetes, and dementia,^32,33^ which may explain the lower dementia-related death risk observed when replacing it with olive oil. The US Food and Drug Administration banned manufacturers from adding partially hydrogenated oils to foods in 2020.^34^ Future studies examining intake of trans-fat–free margarine will be informative. Although the substitution of butter with olive oil was found to be associated with a lower risk of type 2 diabetes, CVD, and total mortality,^11^ we did not find an association with the risk of dementia mortality. Although these previous studies did not examine the associations for butter per se, intake of regular fat dairy products, including cheese, yogurt, and milk, was reported to be either not associated or inversely associated with lower cognitive function, cognitive decline, and dementia.^35,36,37^

Our cohort analyses include several strengths, namely the long follow-up period and large sample size with a high number of dementia death cases. Also, we included genotyping of the *APOE ε4* allele in a large subsample of participants to reduce potential confounding attributed to this well-known risk factor for Alzheimer disease. Additionally, our repeated diet measurements, weight, and lifestyle variables permitted us to account for long-term olive oil intake and confounding factors. Furthermore, the use of dietary cumulative average updates reduced random measurement error by considering within-person variations in intake.

### Limitations

This study has limitations. The possibility of reverse causation cannot be excluded due to the observational nature of our study. However, the 4-year lagged analysis results, consistent with the primary analysis, suggest that olive oil intake is predictive of dementia mortality rather than a consequence of premorbid dementia. While it is plausible that higher olive oil intake could be indicative of a healthier diet and higher SES, our results remained consistent after accounting for the latter. Despite adjusting for key covariates, residual confounding may remain due to unmeasured factors. Also, our study was conducted among health professionals. While this minimizes the potential confounding effects of socioeconomic factors and likely increases reporting due to a high level of education, this may also limit generalizability. Our population was predominantly of non-Hispanic White participants, limiting generalizability to more diverse populations. Additionally, we could not differentiate among various types of olive oil that differ in their polyphenols and other nonlipid bioactive compounds content.

## Conclusions

This study found that in US adults, particularly women, consuming more olive oil was associated with lower risk of dementia-related mortality, regardless of diet quality. Substituting olive oil intake for margarine and mayonnaise was associated with lower risk of dementia mortality and may be a potential strategy to improve longevity free of dementia. These findings extend the current dietary recommendations of choosing olive oil and other vegetable oils to the context of cognitive health and related mortality.

## Notes

Supplement 1.
eMethods.

eResults.
**eTable 1.** Odds Ratios for Dementia-Related Mortality by APOE4 Allelic Dosage **eTable 2.** Risk of Death With Dementia (Composite Outcome) According to Categories of Total Olive Oil **eTable 3.** Joint Associations of Olive Oil Intake and AMED (A), and AHEI (B) With Dementia-Related Mortality Risk **eTable 4.** Risk of Dementia-Related Mortality According to Categories of Total Olive Oil in the Genomic DNA Subsample **eFigure.** Subgroup Analyses for 5g/d Increase in Olive Oil Intake With Dementia-Related Mortality Risk **eTable 5.** Risk of Dementia-Related Mortality According to Categories of Total Olive Oil Without Stopping Diet Update Upon Report of Intermediate Non-Fatal Events **eTable 6.** Risk of Dementia Mortality According to Categories of Total Olive Oil Applying a 4-Year Lag Period Between Dietary Intake and Dementia Mortality **eTable 7.** Risk of Dementia-Related Mortality According to Categories of Total Olive Oil Adjusting for Other Covariates **eTable 8.** Risk of Mortality From Dementia and Other Causes of Death According to Categories of Total Olive Oil Applying a Competing Risk Model
eReferences

Supplement 2.
Data Sharing Statement
