# Errors in Table 1 and Figures

**DOI:** 10.1001/jamanetworkopen.2024.24366

**Published:** 2024-06-25

In the Original Investigation titled “Consumption of Olive Oil and Diet Quality and Risk of Dementia-Related Death,”^1^ published May 6, 2024, there were data errors in the Table 1 row for number of participants in the Health Professionals Follow-up Study, there was a typographical error in the key for panel A of Figure 1, and there were errors in the hazard ratios listed in the text next to the forest plot in panel A of Figure 2. These discrepancies did not affect the results and conclusions of this analysis. This article has been corrected.^1^
