# Supplementary material for: Consumption of Olive Oil and Diet Quality and Risk of Dementia-Related Death
Source: JAMA Netw Open. 2024 May 6;7(5):e2410021. doi: 10.1001/jamanetworkopen.2024.10021 (PMC11074805; doi:10.1001/jamanetworkopen.2024.10021)
Supplement: Supplement 1. — eMethods. eResults. eTable 1. Odds Ratios for Dementia-Related Mortality by APOE4 Allelic Dosage eTable 2. Risk of Death With Dementia (Composite Outcome) According to Categories of Total Olive Oil eTable 3. Joint Associations of Olive Oil Intake and AMED (A), and AHEI (B) With Dementia-Related Mortality Risk eTable 4. Risk of Dementia-Related Mortality According to Categories of Total Olive Oil in the Genomic DNA Subsample eFigure. Subgroup Analyses for 5g/d Increase in Olive Oil Intake With Dementia-Related Mortality Risk eTable 5. Risk of Dementia-Related Mortality According to Categories of Total Olive Oil Without Stopping Diet Update Upon Report of Intermediate Non-Fatal Events eTable 6. Risk of Dementia Mortality According to Categories of Total Olive Oil Applying a 4-Year Lag Period Between Dietary Intake and Dementia Mortality eTable 7. Risk of Dementia-Related Mortality According to Categories of Total Olive Oil Adjusting for Other Covariates eTable 8. Risk of Mortality From Dementia and Other Causes of Death According to Categories of Total Olive Oil Applying a Competing Risk Model eReferences [file jamanetwopen-e2410021-s001.pdf]

Supplemental Online Content

Tessier AJ, Cortese M, Yuan C, et al. Consumption of olive oil and diet quality and risk of dementia-related death. *JAMA Netw Open.* 2024;7(5):e2410021. doi:10.1001/jamanetworkopen.2024.10021

eMethods

eResults

eTable 1. Odds Ratios for Dementia-Related Mortality by APOE4 Allelic Dosage

eTable 2. Risk of Death With Dementia (Composite Outcome) According to Categories of Total Olive Oil

eTable 3. Joint Associations of Olive Oil Intake and AMED (A), and AHEI (B) With Dementia-Related Mortality Risk

eTable 4. Risk of Dementia-Related Mortality According to Categories of Total Olive Oil in the Genomic DNA Subsample

eFigure. Subgroup Analyses for 5g/d Increase in Olive Oil Intake With Dementia-Related Mortality Risk

eTable 5. Risk of Dementia-Related Mortality According to Categories of Total Olive Oil Without Stopping Diet Update Upon Report of Intermediate Non-Fatal Events

eTable 6. Risk of Dementia Mortality According to Categories of Total Olive Oil Applying a 4-Year Lag Period Between Dietary Intake and Dementia Mortality

eTable 7. Risk of Dementia-Related Mortality According to Categories of Total Olive Oil Adjusting for Other Covariates

eTable 8. Risk of Mortality From Dementia and Other Causes of Death According to Categories of Total Olive Oil Applying a Competing Risk Model

eReferences

This supplemental material has been provided by the authors to give readers additional information about their work.

© 2024 Tessier AJ et al. *JAMA Network Open.*

eMethods

Dietary assessment

For other vegetable oils such as corn, safflower, soybean and canola, the consumed amount was calculated based on the brand and type of fat reported by the participants for cooking at home, which included frying, sautéing, baking, and salad dressing. Intake of other fats including margarine and butter was also examined. Total margarine intake was determined from the reported frequency of stick, tub, or soft margarine consumed and based on the amount of margarine used for baking and frying at home. Total butter intake was calculated following the same approach. Intakes of mayonnaise, other fats and nutrients were calculated using the USDA and Harvard University Food Composition Database, [1] and biochemical analyses.

The Alternative Mediterranean index (AMED) score was calculated based on adherence to 9 food items (with either higher or lower intake) that describe the Mediterranean diet: vegetables, legumes, fruits and nuts, whole grains, fish, moderate alcohol, lower intake of red and processed meat, poultry, dairy products and monounsaturated to saturated fatty acids. [2] The modified AMED score ranged from 0-9, a higher score denoting a higher adherence to the Mediterranean diet. The Alternative Healthy Eating Index (AHEI) score was computed based on intake of 11 foods and nutrients that have been positively or inversely associated with chronic disease risk including fruits, vegetables, nuts and legumes, alcohol, red and processed meat, whole grains, sodium, trans-fat, and long-chain omega-3. [3] Each component was scored on a scale from 0 to 10 and the AHEI ranged from 0-110.

Statistical analyses

Given that no heterogeneity was found between cohorts as per the Q-statistics (2.69, p=0.10), pooled risk estimates were obtained by combining data from both cohorts and models were further stratified by cohort. The Laird and DerSimonian fixed-effect inverse variance-weighted approach was also used to meta-analyze the results in a secondary analysis.

We performed several stratified analyses of pre-specified subgroups including age (above vs. below 75 years), living arrangement (alone vs. not alone), BMI (<25 vs. 25-30 vs. ≥30 kg/m^2), family history of dementia (yes vs. no), history of depression (yes vs. no), hypercholesterolemia (yes vs. no), hypertension (yes vs. no), Mediterranean adherence (above vs. below sex-specific AMED median), diet quality (above vs. below sex-specific AHEI median), total vegetable and fruit intake (above vs. below sex-specific median), green vegetable intake (above vs. below sex-specific median), and APOE4 (non-carriers vs. carriers of ≥ 1 allele in the available subset); the cross-product terms were used to test the interaction in multivariable models 3.

Several sensitivity analyses were conducted. First, we ran the primary models using a composite outcome that included participants who reported having dementia during the follow-up and later die, and those with dementia listed on their death certificates. Second, to confirm associations between olive oil intake and dementia mortality independently of the well-recognized APOE4 genetic risk factor, we repeated analyses with further adjustment for APOE4 allele in the available subset. Third, to confirm that we had not introduced a bias by stopping the update of diet upon reported intermediate non-fatal events, we examined the primary associations without stopping the update of diet. Fourth, to minimize the possibility of reverse causation, we repeated the primary analyses with a 4-year lag approach, wherein diet was cumulatively averaged, but time to the outcome began at least 4 years later (e.g., we evaluated whether olive oil intake evaluated through 1994 was associated with fatal dementia risk starting from 1998). Fifth, we further adjusted the main models for personal history of diabetes, a recognized risk factor for dementia; and excluded BMI as a covariate. Lastly, we repeated the primary analysis applying a cause-specific competing risk regression model to estimate the independent association of olive oil intake with dementia mortality and with mortality from other causes.

© 2024 Tessier AJ et al. *JAMA Network Open.*

eResults

In most pre-specified subgroups, we found statistically significant inverse association between olive oil intake and dementia mortality (**eFigure 1, Supplement**). The subgroups in which the association was not significant included having a family history of dementia, living alone, using a multivitamin and not being a carrier of the APOE4 allele. Interactions of subgroup by olive oil intake were not statistically significant except for age, smoking and history of hypercholesterolemia; a stronger inverse association of olive oil intake with dementia mortality was observed in younger participants, in past or current smokers and in participants without hypercholesterolemia. No interaction by AMED or by AHEI was observed.

Sensitivity analyses were consistent with results from primary analyses. From meta-analyzing the cohorts' estimates as opposed to using a pooled dataset, we found the same result (HR: 0.72, 95%CI: 0.64-0.81). No significant heterogeneity between cohorts was noted as per the Q statistics (2.69, p=0.10). Respectively, in the NHS and the HPFS, 745 and 131 participants reported having dementia, but died from another cause. When considering those deaths as outcome cases, results remained similar to those from the primary analysis (**eTable 2, Supplement**). The associations remained comparable and even slightly stronger when not stopping diet update upon report of intermediate non-fatal events such as cancer or CVD (**eTable 5, Supplement**). Additionally, consistent results were observed when applying a 4-year lag between olive oil intake and dementia mortality events (multivariable-adjusted pooled HR for fatal dementia comparing high vs. low intake: 0.72, 95%CI: 0.64-0.81; p-trend: <.0001) (**eTable 6, Supplement**). Results were also consistent when we adjusted for AHEI as opposed to individual food components (pooled HR comparing high vs. low olive oil intake: 0.70, 95%CI: 0.62, 0.79; p-trend: <.0001) and when we excluded BMI from the models (pooled HR comparing high vs. low olive oil intake: 0.70, 95%CI: 0.62-0.78; p-trend: <.0001) (**eTable 7, Supplement**). The competing risk regression models also yielded results consistent results with the primary analysis (**eTable 8, Supplement**). Lastly, when excluding individuals who self-reported having dementia at baseline in 1990 (n=10 in NHS and n=2 in HPFS), results remained identical to the main analysis (data not shown).

© 2024 Tessier AJ et al. *JAMA Network Open.*

eTable 1. Odds ratios for dementia-related mortality by APOE4 allelic dosage.

	NHS			HPFS		
OR (95%CI)	no e4 allele	one e4 allele	two e4 alleles	no e4 allele	one e4 allele	two e4 alleles
dementia-related death	1.0 (ref.)	2.30 (2.05-2.59)	5.42 (4.18-7.02)	1.0 (ref.)	1.74 (1.39-2.18)	9.42 (6.31-14.10)
death with dementia	1.0 (ref.)	2.31 (2.08-2.57)	5.39 (4.26-6.81)	1.0 (ref.)	1.78 (1.44-2.21)	9.08 (6.17-13.40)

NHS, Nurses' Health Study I; HPFS, Health Professionals' Follow-up Study; OR, odds ratio; CI, confidence interval.
ORs are non-adjusted.
The analysis for death with dementia uses the composite outcome that includes participants who reported having dementia during the follow-up and later die, and those with dementia listed on their death certificates.
The analyses were conducted in the genetic subsample (n=27,296).

© 2024 Tessier AJ et al. *JAMA Network Open.*

eTable 2. Risk of death with dementia (composite outcome) according to categories of total olive oil.

	Category of cumulative average olive oil intake					
	**Never or <1/month**	**>0 to ≤ 4.5 g/d (>0 to ≤ 1 tsp)**	**>4.5 to ≤ 7 g/d (>1 tsp to ≤ 0.5 Tbsp)**	**>7 g/d (>0.5 Tbsp)**	P for Trend	HR (95% CI) for 5 g Increase in Olive Oil Intake
**NHS**						
Mean total olive oil	0 +/- 0	1.5 +/- 1.2	5.6 +/- 0.7	11.9 +/- 5.8		
N cases/person-years	**1294**/438566	**2218**/739116	**318**/125884	**387**/182726		
Age-adjusted model 1	1.00 (Ref.)	0.70 (0.65-0.75)	0.70 (0.62-0.79)	0.56 (0.50-0.63)	<.0001	0.85 (0.82-0.89)
MV model 2	1.00 (Ref.)	0.86 (0.80-0.92)	0.89 (0.78-1.01)	0.72 (0.64-0.81)	<.0001	0.91 (0.87-0.94)
MV model 3	1.00 (Ref.)	0.85 (0.79-0.91)	0.86 (0.75-0.97)	0.70 (0.62 0.79)	<.0001	0.89 (0.86-0.93)
**HPFS**						
Mean total olive oil	0 +/- 0	1.5 +/- 1.2	5.6 +/- 0.7	11.3 +/- 5.4		
N cases/person-years	**448**/226931	**752**/381397	**94**/57337	**115**/72138		
Age-adjusted model 1	1.00 (Ref.)	0.83 (0.74-0.94)	0.70 (0.56-0.88)	0.64 (0.52-0.79)	<.0001	0.88 (0.81-0.95)
MV model 2	1.00 (Ref.)	0.97 (0.86-1.09)	0.87 (0.69-1.09)	0.84 (0.68-1.04)	0.07	0.95 (0.88-1.03)
MV model 3	1.00 (Ref.)	0.98 (0.87-1.11)	0.89 (0.71-1.12)	0.87 (0.70-1.08)	0.13	0.97 (0.89-1.04)
**Pooled**						
MV model 3	1.00 (Ref.)	0.88 (0.83-0.93)	0.87 (0.78-0.97)	0.74 (0.66-0.82)	<.0001	0.91 (0.88-0.95)

NHS, Nurses' Health Study I; NHSII, Nurses' Health Study II; HPFS, Health Professionals' Follow-up Study; tsp, teaspoon; tbsp, tablespoon.
The composite outcome used in this analysis includes participants who reported having dementia during the follow-up and later die, and those with dementia listed on their death certificates. All models were stratified by age and time period. Multivariable model 2 was adjusted for Southern European/Mediterranean ancestry (yes, no), married (yes/no), living alone (yes/no), smoking status (never, former, current smoker 1-14 cigarettes/d, 15-24 cigarettes/d, or ≥25 cigarettes/d), alcohol intake (0, 0.1-4.9, 5.0-9.9, 10.0-14.9, and ≥15.0 g/d), physical activity (<3.0, 3.0-8.9, 9.0-17.9, 18.0-26.9, ≥27.0 MET task-h/wk), multivitamin use (yes/no), history of hypertension (yes/no), history of hypercholesterolemia (yes/no), history of diabetes (yes/no), in women postmenopausal status and menopausal hormone use (premenopausal, postmenopausal [no, past, or current hormone use]), total energy intake (kilocalories/d), family history of dementia (yes/no), history of depression (yes/no), census socioeconomic status (9-variable score, in quintiles) and body mass index (<23kg/m2, 23-25 kg/m2, 25-30 kg/m2, 30-35 kg/m2, ≥35kg/m2).

© 2024 Tessier AJ et al. *JAMA Network Open.*

Multivariable model 3 was additionally adjusted for red meat, fruits and vegetables, nuts, soda, whole grains intake (in quintiles), and *trans*-fat. Results were pooled using a pooled dataset of both cohorts and were further stratified by cohort (sex).

© 2024 Tessier AJ et al. *JAMA Network Open.*

eTable 3. Joint associations of olive oil intake and AMED (A), and AHEI (B) with dementia-related mortality risk.

A	Low olive oil (Never or <1/month)	Medium olive oil (>0 to ≤ 0.5 Tbsp or ≤ 7 g/d)	High olive oil (>7 g/d or >0.5 Tbsp)
High AMED (tertile 3)	1.00 (ref)	0.84 (0.75-0.94)	0.66 (0.55-0.78)
Medium AMED (tertile 2)	0.99 (0.86-1.14)	0.82 (0.66-1.01)	0.70 (0.56-0.86)
Low AMED (tertile 1)	0.94 (0.83-1.07)	0.76 (0.57-1.00)	0.72 (0.56-0.93)
**B**			
High AHEI (tertile 3)	1.00 (ref)	0.81 (0.72-0.91)	0.62 (0.52-0.74)
Medium AHEI (tertile 2)	0.94 (0.82-1.08)	0.77 (0.62-0.96)	0.69 (0.56-0.84)
Low AHEI (tertile 1)	0.91 (0.80-1.04)	0.77 (0.59-1.01)	0.73 (0.57-0.95)

AMED, alternative Mediterranean diet index; AHEI, alternative healthy eating index.
This table shows the magnitude of joint associations of olive oil intake and AMED, and AHEI with fatal dementia risk as a complement to Figure 2.
Models were stratified by age, cohort(sex) and calendar time, and adjusted for Southern European/Mediterranean ancestry (yes, no), married (yes/no), living alone (yes/no), smoking status (never, former, current smoker 1-14 cigarettes/d, 15-24 cigarettes/d, or ≥25 cigarettes/d), physical activity (<3.0, 3.0-8.9, 9.0-17.9, 18.0-26.9, ≥27.0 MET task-h/wk), multivitamin use (yes/no), history of hypertension (yes/no), history of hypercholesterolemia (yes/no), history of diabetes (yes/no), in women postmenopausal status and menopausal hormone use (premenopausal, postmenopausal [no, past, or current hormone use]), total energy intake (kilocalories/d), family history of dementia (yes/no), history of depression (yes/no), census socioeconomic status (9-variable score, in quintiles) and body mass index (<23kg/m2, 23-25 kg/m2, 25-30 kg/m2, 30-35 kg/m2, ≥35kg/m2). Results were pooled using a pooled dataset of both cohorts.
AMED score is without monounsaturated/saturated fats intake ratio component.
AHEI score is without polyunsaturated fats intake component.

© 2024 Tessier AJ et al. *JAMA Network Open.*

eFigure. Subgroup analyses for 5g/d increase in olive oil intake with dementia-related mortality risk.

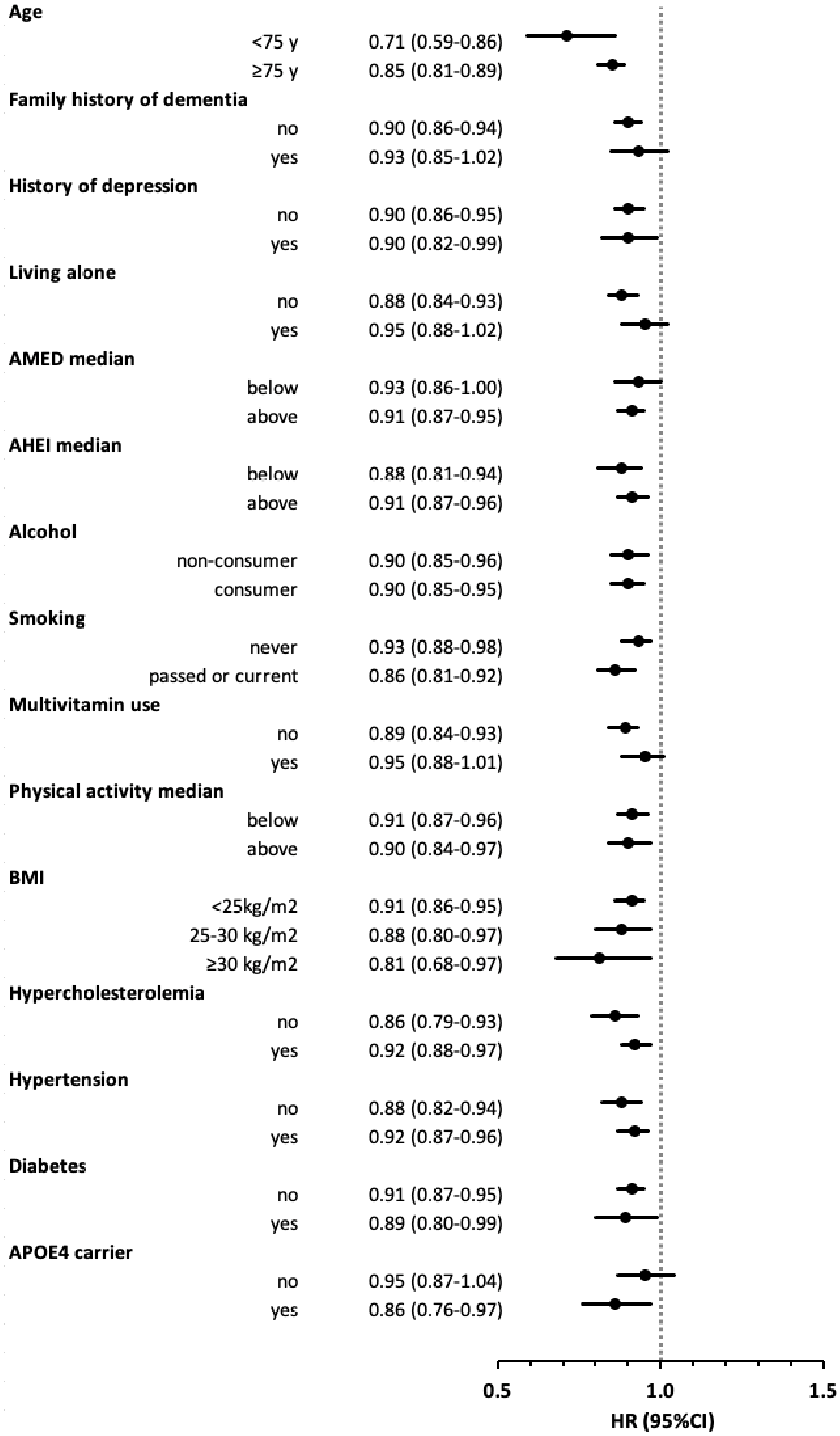

© 2024 Tessier AJ et al. *JAMA Network Open.*

AMED, alternative Mediterranean diet index; AHEI, alternative healthy eating index; BMI, body mass index; HR, hazard ratio; CI, confidence interval. Sex-specific medians were used.
Values are adjusted HR (95% CI). HRs for 5-g increase in olive oil intake in each subgroup category.
Models were stratified by age, cohort(sex) and calendar time, and adjusted for Southern European/Mediterranean ancestry (yes, no), married (yes/no), living alone (yes/no), smoking status (never, former, current smoker 1-14 cigarettes/d, 15-24 cigarettes/d, or ≥25 cigarettes/d), physical activity (<3.0, 3.0-8.9, 9.0-17.9, 18.0-26.9, ≥27.0 MET task-h/wk), multivitamin use (yes/no), history of hypertension (yes/no), history of hypercholesterolemia (yes/no), in women postmenopausal status and menopausal hormone use (premenopausal, postmenopausal [no, past, or current hormone use]), total energy intake (kilocalories/d), family history of dementia (yes/no), history of depression (yes/no), census socioeconomic status (9-variable score, in quintiles) and body mass index (<23kg/m2, 23-25 kg/m2, 25-30 kg/m2, 30-35 kg/m2, ≥35kg/m2). Results are from the pooled dataset.
The APOE4 subgroup analyses were conducted in the genetic subsample (n=27,296).
Significant interaction was found for age (p=0.008), smoking (p=0.025) and hypercholesterolemia (p=0.026).

© 2024 Tessier AJ et al. *JAMA Network Open.*

eTable 4. Risk of dementia-related mortality according to categories of total olive oil in the genomic DNA subsample.

	Category of cumulative average olive oil intake					
	**Never or <1/month**	**>0 to ≤ 4.5 g/d (>0 to ≤ 1 tsp)**	**>4.5 to ≤ 7 g/d (>1 tsp to ≤ 0.5 Tbsp)**	**>7 g/d (>0.5 Tbsp)**	P for Trend	HR (95% CI) per 5 g Increase in Olive Oil Intake
**NHS**						
Mean total olive oil	0 +/- 0	1.5 +/- 1.2	5.6 +/- 0.7	11.8 +/- 5.8		
N cases/person-years	**361**/136976	**677**/228635	**87**/37543	**106**/54188		
Age-adjusted model 1	1.00 (Ref.)	0.73 (0.64-0.83)	0.67 (0.53-0.85)	0.54 (0.43-0.67)	<0.0001	0.85 (0.79-0.92)
MV model 2	1.00 (Ref.)	0.87 (0.76-0.99)	0.84 (0.66-1.06)	0.64 (0.51-0.80)	0.0005	0.89 (0.82-0.96)
MV model 3	1.00 (Ref.)	0.88 (0.77-1.01)	0.81 (0.63-1.03)	0.63 (0.50-0.80)	0.0002	0.88 (0.81-0.95)
**HPFS**						
Mean total olive oil	0 +/- 0	1.5 +/- 1.2	5.6 +/- 0.7	11.4 +/- 5.5		
N cases/person-years	**107**/64607	**208**/113809	**25**/17277	**34**/22574		
Age-adjusted model 1	1.00 (Ref.)	0.82 (0.65-1.04)	0.63 (0.40-0.97)	0.62 (0.42-0.91)	0.01	0.88 (0.76-1.02)
MV model 2	1.00 (Ref.)	0.91 (0.71-1.16)	0.76 (0.48-1.19)	0.78 (0.52-1.17)	0.18	0.96 (0.83-1.11)
MV model 3	1.00 (Ref.)	0.91 (0.71-1.17)	0.77 (0.49-1.22)	0.80 (0.52-1.21)	0.25	0.98 (0.84-1.14)
**Pooled**						
MV model 3	1.00 (Ref.)	0.89 (0.79-1.00)	0.77 (0.62-0.96)	0.66 (0.54-0.81)	<0.0001	0.90 (0.86-0.94)

NHS, Nurses' Health Study I; NHSII, Nurses' Health Study II; HPFS, Health Professionals' Follow-up Study; MV, multivariable, tsp, teaspoon; tbsp, tablespoon.

All models were stratified by age and time period. Model 1 was adjusted for APOE4 allele (carrier vs. non-carrier of at least 1 allele). Multivariable model 2 was further adjusted for Southern European/Mediterranean ancestry (yes, no), married (yes/no), living alone (yes/no), smoking status (never, former, current smoker 1-14 cigarettes/d, 15-24 cigarettes/d, or ≥25 cigarettes/d), alcohol intake (0, 0.1-4.9, 5.0-9.9, 10.0-14.9, and ≥15.0 g/d), physical activity (<3.0, 3.0-8.9, 9.0-17.9, 18.0-26.9, ≥27.0 MET task-h/wk), multivitamin use (yes/no), history of hypertension (yes/no), history of hypercholesterolemia (yes/no), history of diabetes (yes/no), in women postmenopausal status and menopausal hormone use (premenopausal,

postmenopausal [no, past, or current hormone use]), total energy intake (kilocalories/d), family history of dementia (yes/no), history of depression (yes/no), census socioeconomic status (9-variable score, in quintiles) and body mass index (<23kg/m2, 23-25 kg/m2, 25-30 kg/m2, 30-35 kg/m2, ≥35kg/m2). Multivariable model 3 was additionally adjusted for red meat, fruits and vegetables, nuts, soda, whole grains intake (in quintiles), and *trans*-fat. Results were pooled using a pooled dataset of both cohorts and were further stratified by cohort (sex).
The analyses were conducted in the genetic subsample (n=27,296).

eTable 5. Risk of dementia-related mortality according to categories of total olive oil without stopping diet update upon report of intermediate non-fatal events.

	Category of cumulative average olive oil intake					
	**Never or <1/month**	**>0 to ≤ 4.5 g/d (>0 to ≤ 1 tsp)**	**>4.5 to ≤ 7 g/d (>1 tsp to ≤ 0.5 Tbsp)**	**>7 g/d (>0.5 Tbsp)**	P for Trend	HR (95% CI) per 5 g Increase in Olive Oil Intake
**NHS**						
Mean total olive oil	0 +/- 0	1.5 +/- 1.2	5.6 +/- 0.7	11.8 +/- 5.8		
N cases/person-years	**861**/391516	**2054**/771902	**262**/132600	**296**/190273		
Age-adjusted model 1	1.00 (Ref.)	0.61 (0.56-0.66)	0.58 (0.50-0.66)	0.43 (0.38-0.49)	<0.0001	0.81 (0.77-0.85)
MV model 2	1.00 (Ref.)	0.83 (0.76-0.90)	0.81 (0.70-0.93)	0.60 (0.52-0.69)	<0.0001	0.87 (0.83-0.91)
MV model 3	1.00 (Ref.)	0.83 (0.77-0.91)	0.80 (0.69-0.92)	0.59 (0.51-0.68)	<0.0001	0.86 (0.82-0.90)
**HPFS**						
Mean total olive oil	0 +/- 0	1.5 +/- 1.2	5.6 +/- 0.7	11.4 +/- 5.5		
N cases/person-years	**286**/193950	**783**/404473	**96/**61750	**113/**77629		
Age-adjusted model 1	1.00 (Ref.)	0.70 (0.61-0.81)	0.59 (0.47-0.75)	0.52 (0.41-0.65)	<0.0001	0.83 (0.76-0.90)
MV model 2	1.00 (Ref.)	0.92 (0.80-1.06)	0.83 (0.66-1.06)	0.78 (0.62-0.98)	0.03	0.92 (0.85-1.00)
MV model 3	1.00 (Ref.)	0.93 (0.80-1.07)	0.85 (0.67-1.09)	0.79 (0.63-1.01)	0.06	0.93 (0.84-1.01)
**Pooled**						
MV model 3	1.00 (Ref.)	0.83 (0.77-0.89)	0.78 (0.69-0.88)	0.64 (0.56-0.72)	<0.0001	0.88 (0.84-0.92)

NHS,Nurses' Health Study I; NHSII, Nurses' Health Study II; HPFS, Health Professionals' Follow-up Study; MV, multivariable, tsp, teaspoon; tbsp, tablespoon.
All models were stratified by age and time period. Multivariable model 2 was adjusted for Southern European/Mediterranean ancestry (yes, no), married (yes/no), living alone (yes/no), smoking status (never, former, current smoker 1-14 cigarettes/d, 15-24 cigarettes/d, or ≥25 cigarettes/d), alcohol intake (0, 0.1-4.9, 5.0-9.9, 10.0-14.9, and ≥15.0 g/d), physical activity (<3.0, 3.0-8.9, 9.0-17.9, 18.0-26.9, ≥27.0 MET task-h/wk),

multivitamin use (yes/no), history of hypertension (yes/no), history of hypercholesterolemia (yes/no), history of diabetes (yes/no), in women postmenopausal status and menopausal hormone use (premenopausal, postmenopausal [no, past, or current hormone use]), total energy intake (kilocalories/d), family history of dementia (yes/no), history of depression (yes/no), census socioeconomic status (9-variable score, in quintiles) and body mass index (<23kg/m2, 23-25 kg/m2, 25-30 kg/m2, 30-35 kg/m2, ≥35kg/m2). Multivariable model 3 was additionally adjusted for red meat, fruits and vegetables, nuts, soda, whole grains intake (in quintiles), and *trans*-fat. Results were pooled using a pooled dataset of both cohorts and were further stratified by cohort (sex).

eTable 6. Risk of dementia mortality according to categories of total olive oil applying a 4-year lag period between dietary intake and dementia mortality.

	Category of cumulative average olive oil intake					
	**Never or <1/month**	**>0 to ≤ 4.5 g/d (>0 to ≤ 1 tsp)**	**>4.5 to ≤ 7 g/d (>1 tsp to ≤ 0.5 Tbsp)**	**>7 g/d (>0.5 Tbsp)**	P for Trend	HR (95% CI) for 5 g Increase in Olive Oil Intake
**NHS**						
Mean total olive oil	0 +/- 0	1.5 +/- 1.2	5.6 +/- 0.7	11.9 +/- 5.8		
N cases/person-years	**1088**/437732	**1829**/738531	**250**/125833	**305**/182646		
Age-adjusted model 1	1.00 (Ref.)	0.69 (0.64-0.75)	0.66 (0.57-0.75)	0.53 (0.46-0.60)	<.0001	0.83 (0.80-0.87)
MV model 2	1.00 (Ref.)	0.86 (0.80-0.93)	0.85 (0.74-0.98)	0.69 (0.60-0.79)	<.0001	0.89 (0.85-0.93)
MV model 3	1.00 (Ref.)	0.85 (0.79-0.92)	0.82 (0.71-0.95)	0.67 (0.58-0.77)	<.0001	0.88 (0.84-0.92)
**HPFS**						
Mean total olive oil	0 +/- 0	1.5 +/- 1.2	5.6 +/- 0.7	11.3 +/- 5.4		
N cases/person-years	**411**/229917	**678**/386547	**86**/58099	**103/**73065		
Age-adjusted model 1	1.00 (Ref.)	0.83 (0.73-0.94)	0.71 (0.56-0.89)	0.64 (0.51-0.79)	<.0001	0.87 (0.80-0.94)
MV model 2	1.00 (Ref.)	0.96 (0.84-1.09)	0.87 (0.68-1.10)	0.83 (0.66-1.04)	0.08	0.94 (0.87-1.02)
MV model 3	1.00 (Ref.)	0.97 (0.86-1.11)	0.90 (0.71-1.14)	0.86 (0.68-1.08)	0.15	0.95 (0.88-1.04)
**Pooled**						
MV model 3	1.00 (Ref.)	0.88 (0.82-0.94)	0.84 (0.75-0.95)	0.72 (0.64-0.81)	<.0001	0.90 (0.87-0.94)

NHS, Nurses' Health Study I; NHSII, Nurses' Health Study II; HPFS, Health Professionals' Follow-up Study; MV, multivariable, tsp, teaspoon; tbsp, tablespoon.

All models were stratified by age and time period. Multivariable model 2 was adjusted for Southern European/Mediterranean ancestry (yes, no), married (yes/no), living alone (yes/no), smoking status (never, former, current smoker 1-14 cigarettes/d, 15-24 cigarettes/d, or ≥25 cigarettes/d), alcohol intake (0, 0.1-4.9, 5.0-9.9, 10.0-14.9, and ≥15.0 g/d), physical activity (<3.0, 3.0-8.9, 9.0-17.9, 18.0-26.9, ≥27.0 MET task-h/wk), multivitamin use (yes/no), history of hypertension (yes/no), history of hypercholesterolemia (yes/no), history of diabetes (yes/no), in women postmenopausal status and menopausal hormone use (premenopausal, postmenopausal [no, past, or current hormone use]), total energy intake (kilocalories/d), family history of dementia (yes/no), history of depression (yes/no), census socioeconomic status (9-variable score, in quintiles) and

body mass index (<23kg/m2, 23-25 kg/m2, 25-30 kg/m2, 30-35 kg/m2, ≥35kg/m2). Multivariable model 3 was additionally adjusted for red meat, fruits and vegetables, nuts, soda, whole grains intake (in quintiles), and *trans*-fat. Results were pooled using a pooled dataset of both cohorts and were further stratified by cohort (sex).

eTable 7. Risk of dementia-related mortality according to categories of total olive oil adjusting for other covariates.

	Category of cumulative average olive oil intake					
	**Never or <1/month**	**>0 to ≤ 4.5 g/d (>0 to ≤ 1 tsp)**	**>4.5 to ≤ 7 g/d (>1 tsp to ≤ 0.5 Tbsp)**	**>7 g/d (>0.5 Tbsp)**	P for Trend	HR (95% CI) for 5 g Increase in Olive Oil Intake
**NHS**						
Mean total olive oil	0 +/- 0	1.5 +/- 1.2	5.6 +/- 0.7	11.9 +/- 5.8		
N cases/person-years	**1088**/438566	**1829**/739116	**250**/125884	**306**/182726		
MV model 2 + AHEI	1.00 (Ref.)	0.86 (0.79-0.92)	0.84 (0.73-0.97)	0.68 (0.59-0.78)	<.0001	0.89 (0.85-0.93)
MV model 3 excl. BMI	1.00 (Ref.)	0.86 (0.79-0.93)	0.81 (0.70-0.94)	0.67 (0.59-0.77)	<.0001	0.88 (0.84-0.93)
**HPFS**						
Mean total olive oil	0 +/- 0	1.5 +/- 1.2	5.6 +/- 0.7	11.3 +/- 5.4		
N cases/person-years	**407**/226931	**681**/381397	**86**/57337	**104**/72138		
MV model 2 + AHEI	1.00 (Ref.)	0.97 (0.85-1.10)	0.88 (0.70-1.12)	0.86 (0.68-1.07)	0.13	0.95 (0.88-1.03)
MV model 3 excl. BMI	1.00 (Ref.)	0.92 (0.81-1.04)	0.83 (0.65-1.06)	0.75 (0.60-0.95)	0.02	0.92 (0.85-1.00)
**Pooled**						
MV model 2 + AHEI	1.00 (Ref.)	0.86 (0.81-0.92)	0.83 (0.73-0.93)	0.70 (0.62-0.79)	<.0001	0.90 (0.86-0.94)
MV model 3 excl. BMI	1.00 (Ref.)	0.87 (0.82-0.93)	0.82 (0.72-0.92)	0.70 (0.62-0.78)	<.0001	0.89 (0.86-0.93)

NHS, Nurses' Health Study I; NHSII, Nurses' Health Study II; HPFS, Health Professionals' Follow-up Study; tsp, teaspoon; tbsp, tablespoon; AHEI, alternative healthy eating index; BMI, body mass index.
AHEI score is without polyunsaturated fats intake component.
All models were stratified by age and time period. Multivariable model 2 was adjusted for Southern European/Mediterranean ancestry (yes, no), married (yes/no), living alone (yes/no), smoking status (never, former, current smoker 1-14 cigarettes/d, 15-24 cigarettes/d, or ≥25 cigarettes/d), alcohol intake (0, 0.1-4.9, 5.0-9.9, 10.0-14.9, and ≥15.0 g/d), physical activity (<3.0, 3.0-8.9, 9.0-17.9, 18.0-26.9, ≥27.0 MET task-h/wk), multivitamin use (yes/no), history of hypertension (yes/no), history of hypercholesterolemia (yes/no), history of diabetes (yes/no), in women postmenopausal status and menopausal hormone use (premenopausal, postmenopausal [no, past, or current hormone use]), total energy intake (kilocalories/d), family history of dementia (yes/no), history of depression (yes/no), census socioeconomic status (9-variable score, in quintiles) and

body mass index (<23kg/m2, 23-25 kg/m2, 25-30 kg/m2, 30-35 kg/m2, ≥35kg/m2). Multivariable model 3 was additionally adjusted for red meat, fruits and vegetables, nuts, soda, whole grains intake (in quintiles), and *trans*-fat. Results were pooled using a pooled dataset of both cohorts and were further stratified by cohort (sex).

eTable 8. Risk of mortality from dementia and other causes of death according to categories of total olive oil applying a competing risk model.

	Category of cumulative average olive oil intake					
	**Never or <1/month**	**>0 to ≤ 4.5 g/d (>0 to ≤ 1 tsp)**	**>4.5 to ≤ 7 g/d (>1 tsp to ≤ 0.5 Tbsp)**	**>7 g/d (>0.5 Tbsp)**	P for Trend	HR (95% CI) for 5 g Increase in Olive Oil Intake
Dementia-related mortality (n=4,751/37,649 deaths)	1.00 (Ref.)	0.88 (0.83-0.94)	0.84 (0.75-0.95)	0.72 (0.64-0.81)	<.0001	0.91 (0.87-0.94)
Other causes of death (n=32,898/37,649 deaths)	1.00 (Ref.)	0.89 (0.87-0.91)	0.88 (0.84-0.92)	0.83 (0.79-0.86)	<.0001	0.95 (0.93-0.96)

All models were stratified by age, time period and cohort, and adjusted for Southern European/Mediterranean ancestry (yes, no), married (yes/no), living alone (yes/no), smoking status (never, former, current smoker 1-14 cigarettes/d, 15-24 cigarettes/d, or ≥25 cigarettes/d), alcohol intake (0, 0.1-4.9, 5.0-9.9, 10.0-14.9, and ≥15.0 g/d), physical activity (<3.0, 3.0-8.9, 9.0-17.9, 18.0-26.9, ≥27.0 MET task-h/wk), multivitamin use (yes/no), history of hypertension (yes/no), history of hypercholesterolemia (yes/no), history of diabetes (yes/no), in women postmenopausal status and menopausal hormone use (premenopausal, postmenopausal [no, past, or current hormone use]), total energy intake (kilocalories/d), family history of dementia (yes/no), history of depression (yes/no), census socioeconomic status (9-variable score, in quintiles) and body mass index (<23kg/m2, 23-25 kg/m2, 25-30 kg/m2, 30-35 kg/m2, ≥35kg/m2), red meat, fruits and vegetables, nuts, soda, whole grains intake (in quintiles), and *trans*-fat. Results were pooled using a pooled dataset of both cohorts.

eReferences

1. Harvard T.H. Chan School of Public Health. Nutrient Tables. Accessed May 3, 2023. https://www.hsph.harvard.edu/nutrition-questionnaire-service-center/
2. Trichopoulou A, Costacou T, Bamia C, Trichopoulos D. Adherence to a Mediterranean diet and survival in a Greek population. *N Engl J Med*. 2003;348(26):2599-2608. doi:10.1056/NEJMoa025039
3. Chiuve SE, Fung TT, Rimm EB, et al. Alternative dietary indices both strongly predict risk of chronic disease. *J Nutr*. 2012;142(6):1009-1018. doi:10.3945/jn.111.157222
