# Supplementary material for: Consumption of Olive Oil and Diet Quality and Risk of Dementia-Related Death
Source: JAMA Netw Open. 2024 May 6;7(5):e2410021. doi: 10.1001/jamanetworkopen.2024.10021 (PMC11074805; doi:10.1001/jamanetworkopen.2024.10021)
Supplement: Supplement 2. — Data Sharing Statement [file jamanetwopen-e2410021-s002.pdf]

Data Sharing Statement

Tessier. Consumption of Olive Oil and Diet Quality and Risk of Dementia-Related Death. *JAMA Netw Open*. Published May 06, 2024. doi:10.1001/jamanetworkopen.2024.10021

Data
Data available: No

Additional Information
Explanation for why data not available: Data described in the manuscript will not be made publicly available; however, analytic code will be made available at https://github.com/DrTessier. Further information including the procedures for obtaining and accessing data from the Nurses' Health Studies and Health Professionals' Follow-up Study is described at https://www.nurseshealthstudy.org/researchers (email: nhsaccess@channing.harvard.edu) and https://sites.sph.harvard.edu/hpfs/for-collaborators.
